# Supplementary material for: Effect of Different Lead and Cadmium Salts on the Photolytic Degradation of Two Typical Fluoroquinolones under Natural Sunlight Irradiation
Source: Int J Environ Res Public Health. 2022 Dec 25;20(1):323. doi: 10.3390/ijerph20010323 (PMC9819336; doi:10.3390/ijerph20010323)
Supplement: Supplementary file 1 [file ijerph-20-00323-s001.zip › ijerph-2037895-supplementary.pdf]

Supplementary Materials

Table S1. The correlation coefficients (R^2), rate constants (k) and half-life time ($t_{1/2}$) of the two FQs.

FQs	Initial Concentration (mmol/L)	R^2	k (1/h)	$t_{1/2}$ (d)
NOR	0.006	0.959	1.06×10^{-3}	27.4
	0.012	0.973	1.30×10^{-3}	22.2
	0.06	0.961	0.79×10^{-3}	36.7
LVF	0.006	0.929	6.82×10^{-4}	42.3
	0.012	0.912	4.61×10^{-4}	62.6
	0.06	0.518	1.61×10^{-4}	179.8

Table S2. The pseudo-first-order kinetics parameters of FQs at different molar ratios.

Item	0.012 mmol/L NOR				0.012 mmol/L LVF			
	r	k (h^{-1})	$t_{1/2}$ (d)	R^2	r	k (h^{-1})	$t_{1/2}$ (d)	R^2
$Pb(NO_3)_2$	1:1	9.8×10^{-4}	29.6	0.96	1:1	5.7×10^{-4}	51	0.93
$Pb(NO_3)_2$	1:2	1.2×10^{-3}	24.1	0.98	1:2	5.5×10^{-4}	52.9	0.87
$Pb(NO_3)_2$	1:3	1.1×10^{-3}	26.3	0.98	1:3	5.6×10^{-4}	51.9	0.92
$Cd(NO_3)_2$	1:1	1.2×10^{-3}	24.7	0.94	1:1	7.5×10^{-4}	38.4	0.93
$Cd(NO_3)_2$	1:2	1.3×10^{-3}	21.9	0.98	1:2	5.8×10^{-4}	50.1	0.89
$Cd(NO_3)_2$	1:3	1.1×10^{-3}	26.5	0.92	1:3	6.9×10^{-4}	41.7	0.97
$PbCl_2$	1:1	1.3×10^{-3}	22.6	0.97	1:1	6.9×10^{-4}	42.1	0.93
$PbCl_2$	1:2	1.5×10^{-3}	19.8	0.95	1:2	7.6×10^{-4}	38.1	0.93
$PbCl_2$	1:3	1.4×10^{-3}	20.6	0.94	1:3	5.6×10^{-4}	51.4	0.93
$CdCl_2$	1:1	1.3×10^{-3}	23.1	0.96	1:1	8.2×10^{-4}	35.2	0.98
$CdCl_2$	1:2	1.4×10^{-3}	21.1	0.96	1:2	7.9×10^{-4}	36.5	0.96
$CdCl_2$	1:3	1.5×10^{-3}	19.9	0.94	1:3	9.7×10^{-4}	29.7	0.98
Blank	Blank	1.3×10^{-3}	22.0	0.97	Blank	4.4×10^{-4}	65	0.93

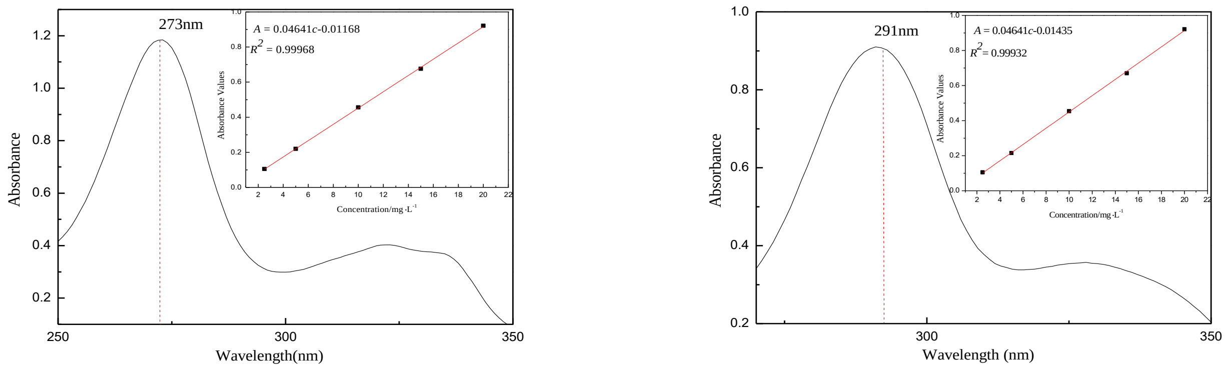

(**a**) (**b**)

Figure S1. UV spectrum of NOR and LVF. (**a**) NOR; (**b**) LVF.

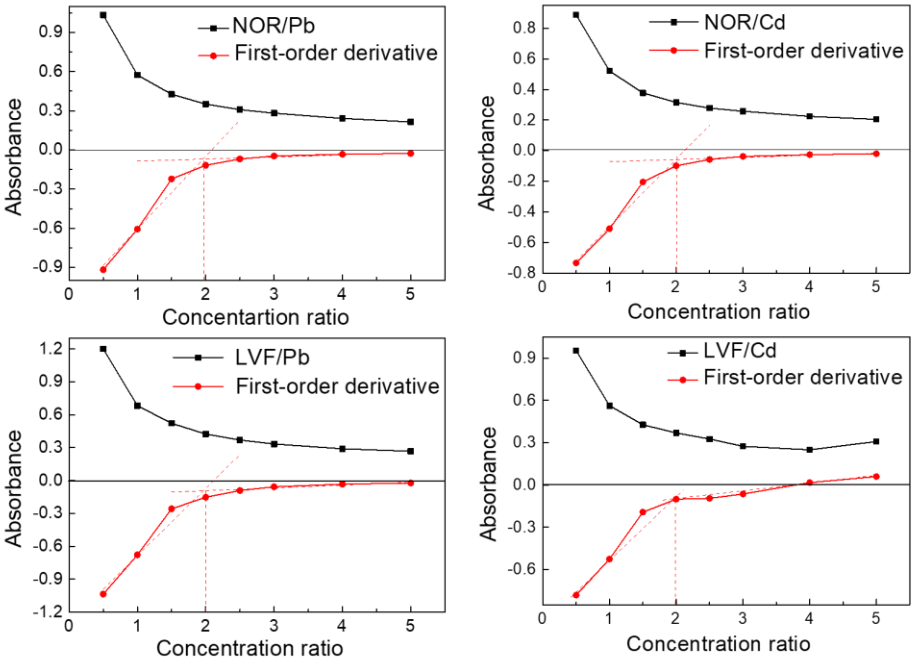

Figure S2. Determination of complexation ratio of NOR/LVF with Pb^{2+} and Cd^{2+}.
